# Graphene-Based Materials for Bone Regeneration in Dentistry: A Systematic Review of In Vitro Applications and Material Comparisons

**DOI:** 10.3390/nano15020088

**Published:** 2025-01-08

**Authors:** Azahara María Narváez-Romero, Francisco Javier Rodríguez-Lozano, María Pilar Pecci-Lloret

**Affiliations:** Dermatology, Stomatology, Radiology and Physical Medicine, Hospital Morales Meseguer, Medicine School, IMIB-Arrixaca, University of Murcia, 30100 Murcia, Spain; azaharanarvaez@gmail.com (A.M.N.-R.); mariapilar.pecci@um.es (M.P.P.-L.)

**Keywords:** graphene, bone regeneration, dentistry, biomaterials

## Abstract

Introduction: Graphene, a two-dimensional arrangement of carbon atoms, has drawn significant interest in medical research due to its unique properties. In the context of bone regeneration, graphene has shown several promising applications. Its robust structure, electrical conductivity, and biocompatibility make it an ideal candidate for enhancing bone tissue regeneration and repair processes. Studies have revealed that the presence of graphene can stimulate the proliferation and differentiation of bone cells, thereby promoting the formation of new bone tissue. Additionally, its ability to act as an effective carrier for growth factors and drugs allows controlled release, facilitating the engineering of specific tissues for bone regeneration. Aim: To assess the efficacy of graphene in enhancing bone regeneration through in vitro studies, identify key safety concerns, and propose directions for future research to optimize its clinical applicability. Materials and methods: The present systematic review was carried out using the PRISMA 2020 guideline. A first search was carried out on 20 November 2023 and was later updated on 14 February and 15 April 2024 in the databases of PubMed, Scopus, and Web of Science. Those in vitro studies published in English that evaluated the potential for bone regeneration with graphene in dentistry and also those which met the search terms were selected. Furthermore, the quality of the studies was assessed following the modified CONSORT checklist of in vitro studies on dental materials. Results: A total of 17 in vitro studies met the inclusion criteria. Among these, 12 showed increased osteoblast adhesion, proliferation, and differentiation, along with notable enhancements in mineralized matrix formation. Additionally, they exhibited a significant upregulation of osteogenic markers such as RUNX and COL1 (*p* < 0.05). However, the variability in methodologies and a lack of long-term assessments were noted as critical gaps. Conclusions: The evaluation of the efficacy and safety of graphene in bone regeneration in dentistry revealed significant potential. However, it is recognized that clinical implementation should be approached with caution, considering identified areas of improvement and suggestions for future research. Future studies should focus on standardized experimental designs, including in vivo studies to evaluate long-term safety, immune responses, and vascularization processes in realistic biological environments.

## 1. Introduction

Bone regeneration in dentistry is a constantly evolving field that, with the aim of restoring the function and aesthetics of affected tissues, has undergone significant advancements in recent decades [1]. Current research in dental bone regeneration is conducted under the premise of the necessity for growth factors, precursor cells, and specific scaffolds such as nanomaterials and nanoparticles, among others [2].

In this context, graphene—a two-dimensional form of carbon with unique properties—has captured the attention of the scientific community due to its high electrical conductivity, mechanical strength, and biocompatibility. Its electrical conductivity enhances cellular communication and mineralization, which are essential for efficient bone healing [3,4]. Previous research has demonstrated that graphene can positively influence cell adhesion and proliferation, as well as the differentiation of bone cells. Moreover, graphene’s capacity to serve as a carrier for growth factors and drugs in tissue regeneration has been the subject of numerous studies. It is important to note that the application of graphene in bone regeneration remains an area of active research, with scientists working to address specific challenges such as the standardization of methodologies, long-term safety, and the optimization of graphene’s properties for medical applications [3,4,5,6].

This material consists of a single layer of carbon atoms organized in a hexagonal lattice, endowing it with remarkable mechanical strength and making it the strongest material ever tested [7,8]. In dentistry, its applications range from dental implants and reconstructive scaffolds to novel drug delivery systems, where durability and biocompatibility are critical [5].

Graphene’s exceptional electrical conductivity and high electron mobility present exciting opportunities for applications in electrical stimulation and biodetection [9]. Such advancements pave the way for personalized approaches to oral surgery, where graphene-based implants or scaffolds should both promote tissue regeneration and monitor its progress. Additionally, graphene-based sensors could improve surgical precision by providing immediate feedback, aiding practitioners in the accurate manipulation of tissues [10]. Beyond its extensive range of applications, graphene’s exceptional mechanical strength, electrical conductivity, and biocompatibility uniquely position it as a transformative material in dentistry, offering significant potential to enhance outcomes in oral surgery, tissue engineering, and the development of durable, antimicrobial dental materials.

Successful bone regeneration in oral surgery depends on the interaction between surgical materials and the biological environment [11]. Research shows that graphene’s biocompatibility supports cell adhesion, proliferation, and differentiation, making it a promising scaffold material for tissue engineering [12]. Graphene scaffolds can accelerate oral tissue bioengineering, bone regeneration, and wound healing by fostering cellular growth [13].

Overall, bone regeneration in dentistry has undergone significant advancements, and the introduction of graphene as a biomaterial has opened new therapeutic possibilities [6,14]. As research progresses, graphene is poised to become a key player in the evolution of bone regeneration techniques in dentistry. However, further research is required to address unresolved challenges, including the long-term safety, reproducibility of results, and optimization of graphene-based technologies for clinical use. Thus, the aim was to evaluate the effectiveness of graphene in bone regeneration and to explore the safety of the use of graphene in dentistry.

## 2. Material and Methods

### 2.1. Statement and Protocol

This systematic review was carried out following the 2020 Preferred Reporting Items for Systematic Reviews and Meta-Analyses (PRISMA) guidelines [15]. Additionally, it has been registered in the Open Science Framework (OSF) registries at https://osf.io/jxqpb (accessed on 5 January 2025).

### 2.2. Inclusion and Exclusion Criteria

The following inclusion criteria were applied in this review with in vitro studies investigating the use of graphene in bone regeneration within the field of dentistry. Exclusion criteria included in vivo studies, experimental and clinical studies, case reports, systematic reviews, narrative reviews, and meta-analyses.

This systematic review adhered to the PICO question:

- PICO Question: Is the use of graphene as a regenerative biomaterial (investigated condition) effective and safe (outcome) compared to bone regeneration without graphene or with other biomaterials distinct from graphene (comparison condition)?
- Structured PICOS Question:
  ▪ Participants (P): Human bone/dental tissues and cells involved in bone regenerative procedures.
  ▪ Intervention or Exposure and Comparison Group (IC): Graphene as a biomaterial in bone regeneration. Comparator: placebo or absence of intervention (not included in the search term combination) or other biomaterials used in bone regeneration, such as hydroxyapatite, calcium phosphates, or synthetic polymer scaffolds.
  ▪ Outcome (O): Bone regeneration.
  ▪ Study (S): In vitro studies.

The reason for focusing strictly on in vitro studies was based on the need to assess only the effects of graphene per se under controlled conditions. This approach ensured sufficient details of its biological interfacing at the cellular membrane level in terms of its adhesion, proliferation, and differentiation capabilities, which act as a backbone for extending such translational research.

### 2.3. Search Strategy

#### 2.3.1. Information Sources

A thorough search was undertaken to identify and analyze articles relevant to this systematic review. The databases used included PubMed, Scopus, and Web of Science. The initial search was conducted on 20 November 2023, and updates were performed on 14 February 2024 and 15 April 2024.

#### 2.3.2. Search Terms

The search covered the period from 1 January 2014 to 15 April 2024, with no language restrictions. The search terms used were derived from the MeSH thesaurus. Boolean operators (“AND”, “NOT”, and “OR”) were employed to combine terms and refine the search strategy.

A total of 191 references were retrieved, distributed as follows: 47 from PubMed, 38 from Scopus, and 106 from Web of Science. A detailed summary of the search terms and corresponding results across the databases is provided in Table 1.

#### 2.3.3. Study Selection

The articles retrieved from the search were imported into the Mendeley reference manager for further selection processing. Initially, duplicate records were removed. Following this, titles and abstracts were reviewed to exclude articles that did not meet the inclusion and exclusion criteria. When the information in the title and abstract was inconclusive, full-text articles were reviewed and analyzed to determine their eligibility.

Finally, all selected studies were thoroughly read and analyzed in full text. Two evaluators (ANR and FJRL) independently reviewed the titles and abstracts to ensure proper selection. Inter-rater reliability achieved was perfect, with a Cohen’s kappa coefficient equal to 1.

#### 2.3.4. Data Extraction

For data extraction, the evaluators (ANR and FJRL) considered the following categories for each relevant study: authors, year of publication, control group, study type, target tissue, type of nanoparticle, observed effects, and type of application. If there was any development of inter-rater disagreements, the procedure followed would involve a standardized discussion between the reviewers in order to establish the source of divergence. In the absence of an agreement, a third assessor (MPPL) analyzed the points of controversy and made a final decision with respect to predefined inclusion criteria and further analysis of the study.

### 2.4. Quality Analysis

The quality of the included studies was assessed using the modified CONSORT (CONsolidated Standards Of Reporting Trials) checklist specifically tailored for in vitro studies of dental materials. This checklist provides guidelines on essential elements that should be reported in preclinical trials, particularly those within the field of dentistry [16]. Two evaluators (ANR and FJRL) independently analyzed the studies and if there were any disagreements, a third evaluator (MPPL) intervened.

The modified CONSORT checklist consists of 14 points encompassing 15 items (with item 2 divided into 2a and 2b), which facilitate the evaluation of the quality of information presented in various sections of an article (abstract, introduction, materials and methods, results, discussion, and additional information).

To evaluate study quality, the following approach was applied:

- Items meeting the necessary conditions were marked YES.
- Items failing to meet these conditions were marked NO.

Studies were then classified based on the percentage of fulfilled items, corresponding to the risk of bias:

- ≥70%: Low risk of bias.
- 50–69%: Moderate risk of bias.
- ≤49%: High risk of bias.

## 3. Results

### 3.1. Study Selection and Flow Diagram

**Figure 1:**
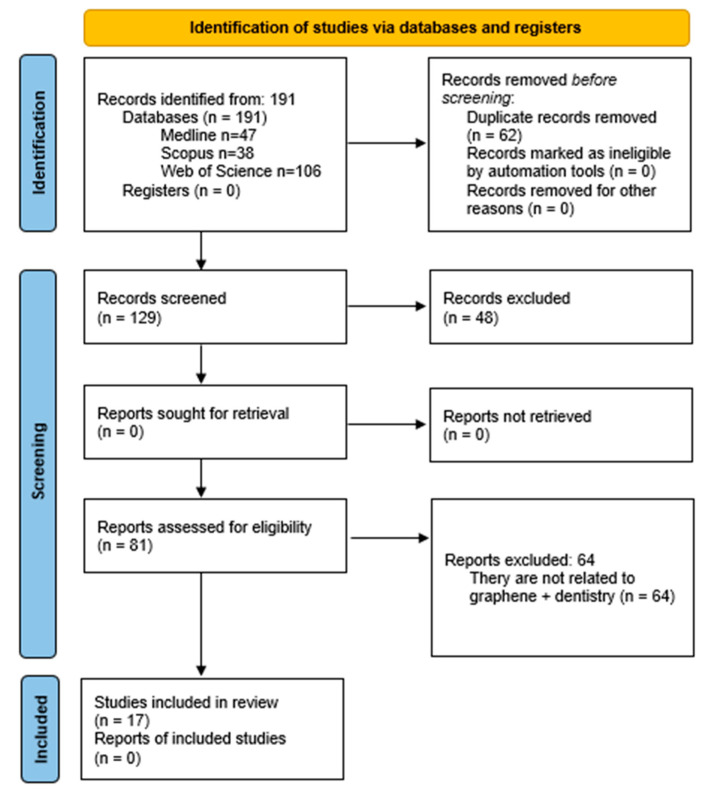
PRISMA 2020 flow diagram. Identification of studies via databases and registers.

Following the bibliographic search, the results were exported to the reference management software Mendeley 1.19.8 (Figure 1). A total of 62 duplicate records were excluded, leaving 129 entries to be screened based on title and abstract. Of these, 45 were excluded for being reviews, two were book chapters, and one was a meeting abstract, resulting in 81 records. Figure 1 provides a PRISMA flow diagram illustrating the screening and selection process. Of the 81 references initially identified, 64 were excluded after screening titles and abstracts. These exclusions were due to irrelevance to the core focus of “graphene + dentistry + bone regeneration”. A more detailed summary is provided here:

1. Non-relevant Topics: excluded studies included those addressing periodontal nanoparticles, preventive dentistry, community dentistry, endodontics, and implantology.
2. Material-Specific Exclusions: studies that investigated graphene as a sealant or exclusively as an antibacterial or angiogenic agent without reference to bone regeneration were excluded.
3. Experimental Contexts: in vitro studies focusing on bone marrow cells without explicit links to dental applications were omitted.
4. Non-Dental Regeneration Sites: research on bone regeneration in non-dental anatomical sites such as the skull, femur, or tibia was excluded.
5. Cell Line Criteria: studies utilizing cell lines like MC3T3-E1, BMSCs, MG63, hMSCs, hADMSCs, or D1 cells unrelated to dentistry were not considered.

Additionally, articles on scaffolds incorporating graphene were excluded if they lacked a connection to dentistry. As a result, 17 articles were selected for full-text reading and comprehensive analysis as the focus of this review.

### 3.2. Data Extraction

The results of the data extraction are presented below in Table 2. This table outlines the previously mentioned characteristics used to evaluate the different studies, thereby enabling their comparison.

### 3.3. Risk of Bias

The assessment of study quality is summarized in Table 2 and was conducted using the modified CONSORT checklist [16] (Table 3). Overall, the studies were rated as having medium to low quality, largely due to most failing to meet the randomization criteria specified in items 6, 7, 8, and 9. These items refer to the method and mechanism of random allocation, which investigator or participant generated it, and who and how blinding was performed following the randomization sequence, respectively. On the other hand, items 2a, 2b, 3, and 13—related to article structuring, group intervention, and funding—were adhered to by all studies. Item 1, based on the structuring of the initial abstract, was not fulfilled by 13 studies. To summarize, one study was at low risk of bias, fifteen studies were at medium low risk of bias, and one study was at high risk of bias.

## 4. Discussion

Graphene (Gr) and its derivatives play a significant role in bone regeneration due to their mechanical and biological properties, especially when applied in bone tissue engineering. This review highlights the advantages of using graphene-based biomaterials in scaffolds for bone tissue engineering. The biological properties of graphene-based biomaterials, as reported in various studies, have demonstrated improvements in osteoblast adhesion and proliferation [34]. However, it is important to critically evaluate the current limitations to better understand graphene’s role in clinical practice.

Some authors, such as Nama et al. [35], advocate for single-examiner data extraction to save time and reduce workload while maintaining sensitivity in systematic reviews. For quality assessment, the CONSORT checklist was modified to evaluate the selected in vitro studies [32], enabling a systematic appraisal of key aspects and potential biases. Other researchers have also employed this guideline for similar studies [36].

Tissue engineering is a rapidly advancing field focused on restoring and replacing human tissues. Each year, a substantial number of individuals require bone replacements, often as a result of dental conditions. Over recent decades, notable advancements in biomedical research, particularly in sophisticated and biocompatible materials, have been achieved [37].

The primary objective of these biomaterials is to support tissue regeneration. Carbon-based nanomaterial scaffolds stand out due to their availability, mechanical strength, and biofunctional properties. These scaffolds promote cellular proliferation, reduce cellular damage, stimulate bone tissue growth, and ensure biocompatibility. Consequently, they are instrumental in forming bone matrices and facilitating cellular interactions essential for bone tissue restoration.

Optimal biomaterials should support bone tissue development in a manner that closely mimics human bone. Graphene and its derivatives exhibit outstanding properties, making them suitable for scaffold use in tissue engineering. Recent studies have expanded the scope of graphene-based materials in biomedical applications, particularly in bone regeneration. These materials demonstrate numerous advantages over other materials, including high surface area, excellent mechanical strength, biocompatibility, biodegradability, gene expression, and the promotion of bone cell proliferation and differentiation [34,38]

There are some limitations in this systematic review, one was the scarcity of published articles specifically addressing graphene-based scaffolds for bone regeneration in dentistry. Many search results pertained to unrelated applications, such as periodontal pathology, preventive dentistry, or scaffolds unrelated to dentistry. Besides that, one of the most relevant limitations seems to be the inexistence of in vivo studies in terms of safety and efficiency after applying graphene for bone regeneration during a long period of time. While in vitro has its value when it refers to cellular or molecular mechanisms, in general, they cannot simulate the conditions of a living system. Thus, immune response feasibility, vascularization, or interaction with the surrounding tissue is quite restricted. Further studies should be focused on developing tests with in vivo conditions under standard conditions. Furthermore, the preparation methodologies of graphene-based scaffolds were quite different in terms of graphene oxide concentration, composite materials, and their fabrication techniques. The variabilities found to date will make it hard to perform direct comparisons among different studies, probably resulting in inconsistent outcomes. Therefore, standardization of scaffold preparation techniques and systematic characterization of their properties are prerequisites to enhancing reproducibility and reliability in graphene research [18].

While the above study has been discussed in detail, it is also very relevant to further explore some of the basic mechanisms involved and the translational challenges that exist with graphene-based biomaterials. Of great interest will be further study on cellular signaling pathways that may mediate the biological effects of graphene. These would be specific points of interaction that may illustrate how graphene works in terms of osteoblast differentiation and deposition of bone matrix; for example, Wnt/β-catenin, TGF-β, and BMP signaling pathways. It could also involve modulation of the immune microenvironment through the polarization of macrophages and/or the release of cytokines [19,38].

However, translating these findings into clinical practice faces significant challenges. While the scalability issues have been touched upon, there is a serious need to understand the more pragmatic know-how to respond properly to the economic viability and barrier issues involved in graphene-based technologies. Producing high-quality graphene in quantity is currently an important challenge because the chemical vapor deposition and chemical exfoliation methods involve manufacturing with high costs and low yields. Innovations involving green synthesis techniques and recycling processes may reduce production cost while sustaining material quality [39].

Furthermore, batch-to-batch consistency and attainment of medical-grade standards in graphene determine further expansion of graphene-based clinical developments. The translational barrier to the integration of graphene-based materials into conventional clinical workflows is another important development. The development has to devise ways of producing graphene-enabled scaffolds or coating compatible with the current approaches without losing key mechanical and biological properties in translation. Practical strategies that take up such translational challenges by composite material design that marries graphene to biodegradable polymers or ceramics appear particularly desirable [40]. Finally, there is the economic feasibility issue. The real cost of graphene-based biomaterials compared with traditional alternatives, along with their durability and performance over time in the oral environment, will provide the basis for translation into clinical applications. Therefore, these current challenges can be overcome only by collaboration between researchers, industrial stakeholders, and policy makers in fully exploiting graphene for regenerative medicine [41].

## 5. Conclusions

Our systematic review suggests the efficacy of graphene in promoting in vitro bone regeneration. Specifically, 12 of the 17 included studies reported significant enhancements in osteo-genic differentiation, with configurations such as graphene oxide (GO) composites showing increased ALP activity, RUNX2 expression, and mineralized matrix formation.

- The review has addressed concerns regarding the safety of graphene in dental settings. Graphene’s biocompatibility, coupled with its ability to enhance cellular interactions and reduce inflammation, highlights its suitability for regenerative applications.
- Composite materials, such as GO-poly-L-lactic acid (PLLA) scaffolds and GO/hydroxyapatite (HA) blends, have emerged as some of the most promising configurations for further exploration.

Future studies should focus on standardized experimental designs, including in vivo studies to evaluate long-term safety, immune responses, and vascularization processes in realistic biological environments. Clinical trials should assess specific graphene-based scaffolds, such as GO/PLLA or GO/HA composites, for their ability to replicate in vitro findings in human applications. Furthermore, optimizing the cost and scalability of graphene production will be critical for advancing its clinical feasibility.

## Figures and Tables

**Table 1:** Search strategy.

Database	Search Terms	Results
	1# graphene AND dent* AND regen*	142
	2# graphene AND dent* AND regen* AND bone	76
Pubmed	3# graphene AND dent* AND regen* AND (bone OR osteo*)	82
	4# graphene AND dent* AND regen* AND (bone OR osteo*) NOT implant*	**47**
	1# graphene AND dent* AND regen*	119
Scopus	2# graphene AND dent* AND regen AND bone	85
	3# graphene AND dent* AND regen* AND (bone OR osteo*)	92
	4# graphene AND dent* AND regen* AND (bone OR osteo*) NOT implant*	**38**
	1# graphene AND dent* AND regen*	290
	2# graphene AND dent* AND regen* AND bone	165
Web of Science	3# graphene AND dent* AND regen* AND (bone or osteo*)	182
	4# graphene AND dent* AND regen* AND (bone or osteo*) NOT implant	**106**

**Table 2:** Summary of the various parameters of the selected studies for data extraction.

**Author**	**Objectives/Generalities**	**Material and Methods**	**Conclusions**
Qiu, Z. et al., 2024 [17]	Dental pulp stem cells (DPSCs), capable of osteogenic differentiation under suitable conditions, require effective support materials for 3D tissue fabrication and bone regeneration. This study investigates the biological performance of human DPSCs on composite scaffolds made of graphene oxide (GO) and poly-L-lactic acid (PLLA).	-Cell line: hDPSCs -Scaffolds: 3D GO/PLLA scaffolds containing 0.15 wt%, 0.20 wt%, and 0.25 wt% GO	The data demonstrate that GO and PLLA integrate successfully, with GO/PLLA scaffolds exhibiting excellent bioactivity and biocompatibility with DPSCs. The 0.15% GO/PLLA scaffold significantly promoted DPSC proliferation and osteogenic differentiation, as shown by increased calcium nodule formation, enhanced ALP activity and staining, and upregulated expression of RUNX2 and COL1. These findings underscore the potential of GO/PLLA scaffolds as a promising material for cell culture and oral bone tissue engineering.
López-García, S. et al., 2024 [18]	To evaluate silk fibroin, with and without graphene, as a scaffold material for regenerative endodontics.	-Cell line: hDPSCs -Scaffolds: three types of scaffolds were studied. (1) Pure fibroin materials without incorporated GO designated as “SF”, (2) SF scaffolds containing GO adsorbed on their surface were termed as “SF/GO”, and (3) those in which the superficially adsorbed GO was reduced were termed “SF/rGO”.	The data indicate that SF/GO and SF/rGO scaffolds promote hDPSC differentiation by upregulating key osteo/odontogenic markers and facilitating extracellular matrix mineralization. However, further in vivo studies are required to validate their potential.
Li, Y. et al., 2024 [19]	Polydopamine (PDA) was used to reduce GO, forming the PDA-GO complex, which was combined with chitosan (CS) to develop PDA-GO/CS composite scaffolds for bone tissue engineering. PDA-GO improved the degradation rate of CS. The scaffolds’ physicochemical and antimicrobial properties were evaluated to determine the optimal composition, while biocompatibility was assessed using phalloidin and live/dead staining. Osteogenic differentiation of human dental pulp stem cells (hDPSCs) was analyzed via ALP staining, RT-qPCR, and alizarin red S staining.	-Cell line: hDPSCs -Scaffolds: A:CS;B:0.1%PDA-GO/CS;C:0.3%PDA-GO/CS;D:0.5%PDA-GO/CS;E:0.7%PDA-GO/CS -Biocompatibility and osteogenic assays: 0.3%PDA-GO/CS extracts.	The results confirmed the successful synthesis of the PDA-GO compound and its integration into PDA-GO/CS composite scaffolds. The 0.3% PDA-GO/CS scaffold showed improved antibacterial activity and hydrophilicity while reducing CS’s degradation rate. *In vitro*, it demonstrated excellent biocompatibility, promoting early hDPSC proliferation, migration, and osteogenic differentiation. These findings highlight PDA-GO/CS as a promising scaffold material for cell culture and bone tissue engineering applications.
Qasim, S. et al., 2023 [20]	Membranes are crucial for treating periodontal defects and guided bone regeneration. This study synthesized chitosan-based membranes with nanographene oxide, hydroxyapatite (HA), and chlorhexidine digluconate (CHX) via solvent casting. Four types (CH, CCG, 3511, and 3322) were evaluated for physicochemical, mechanical, barrier, degradation, and antimicrobial properties.	Scaffolds: four membranes. Chitosan (CH), CH/CHX/GO were used in a ratio of 50:25:25 (CCG). HA-based membranes were synthesized at a ratio of CH/HA/CHX/GO: 30:50:10:10 (CHCG 3511) and 30:30:20:20 (CHCG 3322). -No biocompatibility assays	The membranes demonstrated suitable mechanical properties and handling in both dry and wet conditions for clinical use. Chemical analysis indicated strong functional group interactions, while the nanocomposites’ unique physicochemical properties created an efficient antimicrobial system. Degradation studies, drug release profiles, and antimicrobial results suggest that 3511 could serve as a surface layer in a functionally graded membrane for guided bone regeneration.
Souza, A. et al., 2022 [21]	This study aimed to synthesize and characterize polymeric scaffolds of chitosan/xanthan gum/graphene oxide-hydroxyapatite (HA-GO) nanocomposites combined with mesenchymal stem cells for regenerative dentistry.	-Scaffolds: The chitosan-xanthan gum complex (CX) was blended with HA-GO at varying graphene oxide (GO) concentrations (0.5 wt%, 1.0 wt%, 1.5 wt%). Scaffolds were characterized using XRD, FTIR, Raman spectroscopy, TGA, SEM, and contact angle measurements. Mechanical properties were evaluated via compressive strength testing, and *in vitro* bioactivity and cytotoxicity (MTT assay) were assessed. Data were analyzed for normality and homogeneity. -Cell line: hDPSCs	XRD confirmed HA peaks, and FT-IR identified CX functional groups. Raman verified GO quality, while TGA showed CX degradation. SEM revealed porous scaffolds with HA adhesion. Hydrophilicity varied, with CXHA being most hydrophilic (*p* < 0.05). GO at 1.0 wt% enhanced compressive strength. Bioactivity tests showed apatite formation, and MTT assays indicated high cell viability in CXHAGO 1.0% and 1.5%. CXHAGO scaffolds demonstrate excellent properties for regenerative dentistry.
Daulbayev, C. et al., 2022 [22]	This article details the fabrication and characterization of an electrospun composite scaffold made from graphene oxide (GO), calcium hydroxyapatite (HAp), and polycaprolactone (PCL). PCL offers excellent medical properties and chemical resistance, while GO and HAp provide superior biocompatibility, mechanical strength, and conductivity. Synthesized from biowaste materials, the GO/HAp composite was incorporated into biodegradable PCL to develop a scaffold designed to enhance osteogenesis and osteoblast differentiation for medical applications.	-Scaffolds: GO, wt. % (0.01;0.05; 0,1; 0.5; 1.0) HAP, PCL and C_2_H_5_OH. -Cell Line: MC3T3-E1	The GO/HAp/PCL composite scaffold demonstrates excellent biocompatibility and antimicrobial properties, making it a promising matrix for bone tissue regeneration and applications in medicine and clinical dentistry.
Ferroni, L. et al., 2022 [23]	Bone regeneration in dentistry requires osteoinductive, antibacterial biomaterials. Polycaprolactone (PCL) combined with reduced graphene oxide (rGO) was optimized in this study to create a biocompatible, antibacterial surface supporting MSC adhesion and differentiation. Composites with three rGO concentrations were tested.	Scaffolds: Pure PCL (Sigma-Aldrich, St. Louis, MO, USA) was mixed with rGO (Abalonyx AS, Oslo, Norway) at the percentages of 1.6% *w*/*w*, 3% *w*/*w*, or 5% *w*/*w* via melt compounding strategy assisted by a twin screw extruder (Themo Fisher Scientific, Waltham, MA, USA). -Cell line: 1. NCTC clone 929 (mouse fibroblast cell line; ATCC) 2. Human adipose-derived mesenchymal stem cells	The 5% rGO-PCL composite showed the highest bacteriostatic activity against Gram-positive bacteria and excellent biocompatibility. MSCs adhered, proliferated, and expressed extracellular matrix components more effectively on this surface. It also demonstrated superior osteoinductive properties, with increased alkaline phosphatase activity, mineralized matrix deposition, and osteogenic marker expression. These results highlight the potential of 5% rGO-PCL for bone tissue engineering.
Ketkar, G. et al., 2022 [24]	Conventional nanoparticle synthesis often involves toxic stabilizers, posing environmental risks. This study focused on the “green synthesis” of copper nanoparticles reinforced with graphene oxide and amla extract, evaluating their cytotoxicity. Copper’s antibacterial properties and graphene oxide’s structural strength make them ideal for nanocomposites. The objective was to develop an eco-friendly nanocomposite and assess its cytotoxicity.	Nanocomposite synthesis was achieved by mixing 50 mL of both 1M solutions of copper and graphene oxide nanoparticles. Cell Line: data not available	The copper and graphene oxide nanocomposite is safe for dental applications at concentrations up to 20 µL. Cytotoxic effects were noted at 40 µL, with significant toxicity at 80 µL. Within these limits, the nanocomposite is suitable for use as a periodontal dressing or in bone grafts for periodontal regeneration.
Mancinelli, R. et al., 2021 [25]	Equine bone blocks are biocompatible and osteoconductive, supporting bone regeneration and biomineralization when combined with hDPSCs, which differentiate into osteoblasts. In this study, collagenated equine bone blocks were coated with ammonia-functionalized graphene oxide (G-N) at 2 μg/mL (G-N2) and 10 μg/mL (G-N10). Raman spectroscopy confirmed GN coating homogeneity, and TGA measured GN deposition. The study aimed to evaluate *in vitro* the effect of GN-coated equine bone blocks with melatonin on hDPSC proliferation and differentiation.	Scaffolds: collagenated equine bone blocks were coated with ammonia-functionalized graphene oxide (G-N) at 2 μg/mL (G-N2) and 10 μg/mL (G-N10). Cell line: hDPSCs	Equine bone blocks, known for their osteogenic, biocompatible, and osteoconductive properties, support bone regeneration and biomineralization, particularly with hDPSCs, which differentiate into osteoblasts. This study coated collagenated equine bone blocks with ammonia-functionalized graphene oxide (G-N) at 2 μg/mL (G-N2) and 10 μg/mL (G-N10). Raman spectroscopy verified coating homogeneity, and TGA quantified GN deposition. The objective was to evaluate *in vitro* the impact of GN-coated equine bone blocks with melatonin on hDPSC proliferation and differentiation.
García-Contreras, R. et al., 2021 [26]	This study evaluated the cytotoxicity and cell proliferation of graphene oxide (GO) in cultures of gingival fibroblasts, dental pulp cells, and human osteoblasts, along with the physical, mechanical, and biological properties of GO-enriched polymethyl methacrylate (PMMA). GO was characterized via SEM, while cytotoxicity and proliferation were assessed using the MTT bioassay. Physical and mechanical properties, including flexural strength and elastic modulus, were measured with a universal testing machine. Adsorption, solubility, and porosity were analyzed via weight measurements and visual inspection.	-Scaffolds: The GO-enriched acrylic samples were prepared with a 50 ± 1 × 15 ± 1 × 3.0 ± 0.1 mm dimension. -Cell line: human gingival fibroblasts (HGF), human dental pulp cells (HPC), and human osteoblasts (HBC).	Graphene oxide (GO) displays a heterogeneous morphology with a particle size of 66.67 ± 64.76 μm and shows slight to no cytotoxicity (>50–75% cell viability) over 1–30 days. GO-enriched PMMA significantly enhances osteoblast proliferation (45 ± 8.2%, *p* < 0.01) after 24 h. PMMA with GO also improves physical and mechanical properties without affecting sorption, solubility, or porosity. In conclusion, GO, alone or in PMMA, demonstrates good biocompatibility, promotes cell proliferation and regeneration, and enhances PMMA’s material properties.
Lee, J. et al., 2018 [27]	This study prepared dicalcium phosphate (DCP) composites coated with reduced graphene oxide (rGO) at concentration ratios of 5:2.5, 5:5, and 5:10 g/mL (DCP-rGO composites) and analyzed their physicochemical properties.	Scaffolds: composites coated with reduced graphene oxide (rGO) at concentration ratios of 5:2.5, 5:5, and 5:10 g/mL Cell line: MC3T3-E1 preosteoblasts	This study suggests that hybrid DCP-rGO composites may be potent factors in accelerating bone tissue regeneration.
Vera-Sánchez, et al., 2016 [28]	This study explored various combinations of graphene and silk fibroin constructs for bioengineering applications in regenerative dentistry, focusing on their interaction with human periodontal ligament stem cells (hPDLSCs).	Scaffolds: The films were named as GO:SF (ratio expressed in dry weight). GO:SF (3:1) films were produced using a mixture of 750 mL of GO water dispersion (4 mg/mL) and 66.7 mL of SF water solution (1.5% *w*/*v*) leading to the formation of films composed of 3 mg of GO and 1 mg of SF. GO:SF (1:1) films were made by means of evaporation of 500 mL of GO water dispersion (4 mg/mL) mixed with 133.3 mL of SF water dissolution (1.5% *w*/*v*), being the final composition of each film, 2 mg of GO and 2 mg of SF. A blend of 250 mL of GO water dispersion (4 mg/mL) and 200 mL of SF water solution (1.5% *w*/*v*) was evaporated to produce GO:SF (1:3) films, whose composition was 1 mg of GO and 3 mg of SF per film. -Cell line: hPDLSCs	The results offer valuable insights for designing structures that support effective cell grafting, maintain cell viability and proliferation, promote spontaneous osteoblastic differentiation, and significantly enhance physiological cementum synthesis compared to existing biomaterials.
Golzar, H. et al., 2020 [29]	This study developed 3D-printed β-TCP scaffolds filled with a lyophilized gelatin/reduced graphene oxide/magnesium/arginine (GRMA) matrix to enhance DPSC osteogenic potential. RMA concentrations (0, 0.25%, 0.75%) were evaluated for effects on scaffold morphology, mechanics, and biological activity.	-Scaffold: hybrid constructs consisted of 3D-printed Beta-tricalcium phosphate (β-TCP)-based scaffolds filled with freeze-dried gelatin/reduced graphene oxide/magnesium/arginine (GRMA). -Cell line: DPSCs	The results show that incorporating RMA into scaffolds enhances mechanical properties and promotes cell proliferation and differentiation. The β-TCP/0.25 GRMA scaffold demonstrated the highest ALP activity, cell proliferation, and upregulation of bone-related genes and proteins (COL-I, RUNX2, OCN) after 14 days. Thus, β-TCP/0.25 GRMA scaffolds are promising candidates for bone tissue engineering.
Zhang, W. et al., 2017 [30]	This study introduces a novel “growth factor-immobilized cell sheet” design controlled magnetically using Fe_3_O_4_ magnetic nanoparticles coated with nanoscale graphene oxide (nGO@Fe_3_O_4_). These nanoparticles effectively label dental pulp stem cells (DPSCs) without compromising cell viability, enabling controlled delivery of growth factors.	Scaffolds: nanoparticles coated with nanoscale graphene oxide (nGO@Fe_3_O_4_) Cell line: hDPSCs	The results highlight the promising potential of cell sheet technology as an innovative tissue engineering approach for future regenerative medicine applications.
Zhou, Q. et al., 2016 [31]	This study evaluated the *in vitro* bioactivity of human periodontal ligament stem cells (PDLSCs) on a graphene oxide-coated titanium substrate (GO-Ti) compared to a sodium titanate substrate (Na-Ti).	Scaffolds: graphene oxide-coated titanium substrate (GO-Ti) compared to a sodium titanate substrate (Na-Ti). Cell line: PDLSCs	The results indicate that combining graphene oxide (GO) with periodontal ligament stem cells (PDLSCs) offers a promising approach for regenerative dentistry.
Lee, J. et al., 2015 [32]	This study investigated the potential of reduced graphene oxide (rGO) and hydroxyapatite (HAp) nanocomposites (rGO/HAp NC) to enhance osteogenesis in MC3T3-E1 preosteoblasts and stimulate new bone formation.	Scaffolds: rGO/HAp NC Cell line: MC3T3-E1 preosteoblasts	The results suggest that rGO/HAp nanocomposites (NCs) hold promise for developing dental and orthopedic bone grafts, leveraging their ability to stimulate osteogenesis and accelerate bone regeneration.
Rodríguez-Lozano, F.J. et al., 2014 [33]	This study aimed to examine the effects of graphene oxide (GO), silk fibroin (SF), and GO/SF composite films on the mesenchymal phenotype, viability, adhesion, and proliferation of periodontal ligament stem cells (PDLSCs).	Scaffolds: films of GO, SF, and GO/SF Cell line: PDLSCs	The results indicate that bioengineered constructs combining human dental stem cells, silk fibroin, and graphene oxide (GO) show significant potential for regenerative dentistry applications.

**Table 3:** Risk of bias.

	1	2a	2b	3	4	5	6	7	8	9	10	11	12	13	14	Risk of Bias
Qiu Z et al., 2024 [17]	✓	✓	✓	✓	✓	✓	✕	✕	✕	✕	✓	✓	✓	✓	✓	73.3%
López-García S et al., 2024 [18]	✓	✓	✓	✓	✓	✓	✕	✕	✕	✕	✓	✓	✓	✓	✕	66.6%
Li Y et al., 2024 [19]	✕	✓	✓	✓	✓	✓	✕	✕	✕	✕	✓	✓	✕	✓	✓	60%
Qasim S et al., 2023 [20]	✕	✓	✓	✓	✓	✓	✕	✕	✕	✕	✓	✓	✕	✓	✕	53.3%
Souza A et al., 2022 [21]	✕	✓	✓	✓	✓	✓	✕	✕	✕	✕	✓	✓	✕	✓	✕	53.3%
Ferroni L et al., 2022 [23]	✕	✓	✓	✓	✓	✓	✕	✕	✕	✕	✓	✓	✕	✓	✕	53.5%
Daulbayev C. et al., 2022 [22]	✕	✓	✓	✓	✓	✓	✕	✕	✕	✕	✕	✕	✕	✓	✕	40%
Ketkar G et al., 2022 [24]	✓	✓	✓	✓	✓	✓	✕	✕	✕	✕	✕	✕	✓	✓	✕	53.3%
García-Contreras R et al., 2021 [26]	✓	✓	✓	✓	✓	✓	✕	✕	✕	✕	✓	✓	✓	✓	✕	66.6%
Mancinelli R et al., 2021 [25]	✕	✓	✓	✓	✓	✓	✕	✕	✕	✕	✓	✓	✕	✓	✓	60%
Golzar H et al., 2020 [29]	✕	✓	✓	✓	✓	✓	✕	✕	✕	✕	✓	✓	✕	✓	✕	53.3%
Lee J et al., 2018 [27]	✕	✓	✓	✓	✓	✓	✕	✕	✕	✕	✓	✓	✕	✓	✕	53.3%
Zhang W et al., 2017 [30]	✕	✓	✓	✓	✓	✓	✕	✕	✕	✕	✓	✓	✕	✓	✓	60%
Vera-Sánchez M et al., 2016 [28]	✕	✓	✓	✓	✓	✓	✕	✕	✕	✕	✓	✓	✕	✓	✕	53.3%
Zhou Q et al., 2016 [31]	✕	✓	✓	✓	✓	✓	✕	✕	✕	✕	✓	✓	✕	✓	✓	60%
Lee J et al., 2015 [32]	✕	✓	✓	✓	✓	✓	✕	✕	✕	✕	✓	✓	✕	✓	✕	60%
Rodriguez-Lozano FJ et al., 2014 [33]	✕	✓	✓	✓	✓	✓	✕	✕	✕	✕	✓	✓	✕	✓	✕	53.3%

## Data Availability

Not applicable.
